# Anasarca as the presenting symptom of juvenile dermatomyositis: a case series

**DOI:** 10.1186/s12969-021-00604-3

**Published:** 2021-08-13

**Authors:** Emily E. Schildt, Deirdre De Ranieri

**Affiliations:** Department of Rheumatology, Ann & Robert H. Lurie Children’s Hospital of Chicago, Chicago, IL United States

**Keywords:** Juvenile dermatomyositis, Anasarca, Generalized edema, Vascular permeability, Muscle enzymes, Myositis, Immunosuppression

## Abstract

**Background:**

Juvenile Dermatomyositis (JDM) is an autoimmune disease that typically presents with classic skin rashes and proximal muscle weakness. Anasarca is a rare manifestation of this disease and is associated with a more severe and refractory course, requiring increased immunosuppression. Early recognition of this atypical presentation of JDM may lead to earlier treatment and better outcomes.

**Case presentation:**

We present two female patients, ages 11 years old and 4 years old, who presented to the ED with anasarca and were subsequently diagnosed with JDM. Both patients required ICU-level care and significant immunosuppression, including prolonged courses of IV methylprednisolone, IVIG, and Rituximab.

**Conclusions:**

Anasarca is a rare presentation of Juvenile Dermatomyositis, but it is important for clinicians to recognize this manifestation of the disease. Early recognition and treatment will lead to better outcomes in these children and hopefully decrease the need for prolonged hospitalization and ICU level care.

## Background

Juvenile dermatomyositis (JDM) is the most common chronic inflammatory myopathy of childhood. It is an autoimmune disease that primarily affects the muscles and skin but can also affect the blood vessels, manifesting as localized edema. Although presentations of JDM can be variable, symptoms typically include proximal muscle weakness and classic skin rashes such as Gottron’s papules and heliotrope rash. Localized periorbital edema and extremity swelling have also been described in patients with JDM. However, anasarca, which is a severe and generalized form of edema, is a very uncommon presentation of JDM and has been described in only a few case reports of children with JDM [1–12]. In these cases, the diagnosis of JDM was confirmed by imaging, pathology, and/or laboratory studies.

We describe two patients with a final diagnosis of Juvenile Dermatomyositis who presented with anasarca, both of whom required ICU-level care. Anasarca is a rare presentation of this disease but can be life-threatening, and expedient identification and treatment is crucial.

### Case 1

An 11-year-old girl presented to our ED with a 2 week history of generalized swelling, diffuse myalgia, and generalized weakness and. She was admitted for further workup and management. Her past medical history was significant for RF-negative Polyarticular Juvenile Idiopathic Arthritis diagnosed at age 8 and involving her bilateral wrists and fingers and successfully treated with daily naproxen and two rounds of intra-articular corticosteroid injections [13]. Her second course of intraarticular corticosteroid injections was completed 3 months prior to presentation.

Her initial physical examination was significant for 2+ pitting edema of her lower legs bilaterally as well as non-pitting edema of her bilateral thighs, arms, and face. She had no arthritis at this time. She was noted to have hypopigmentation on her right eyelid and cheek. She had no evidence of Gottron’s papules or a heliotrope rash. She was noted to be very weak with inability to ambulate, sit unsupported, or support her head. She described her limbs as feeling “heavy.” She had dysphonia, but gag reflex was not tested at that time. Review of her growth chart was notable for a 4 kg weight gain over the past month. Physical therapy evaluation was significant for Manual Muscle Test 8 (MMT8) score of 40/80 and Childhood Myositis Assessment Scale (CMAS) score of 2/52.

**Fig. 1:**
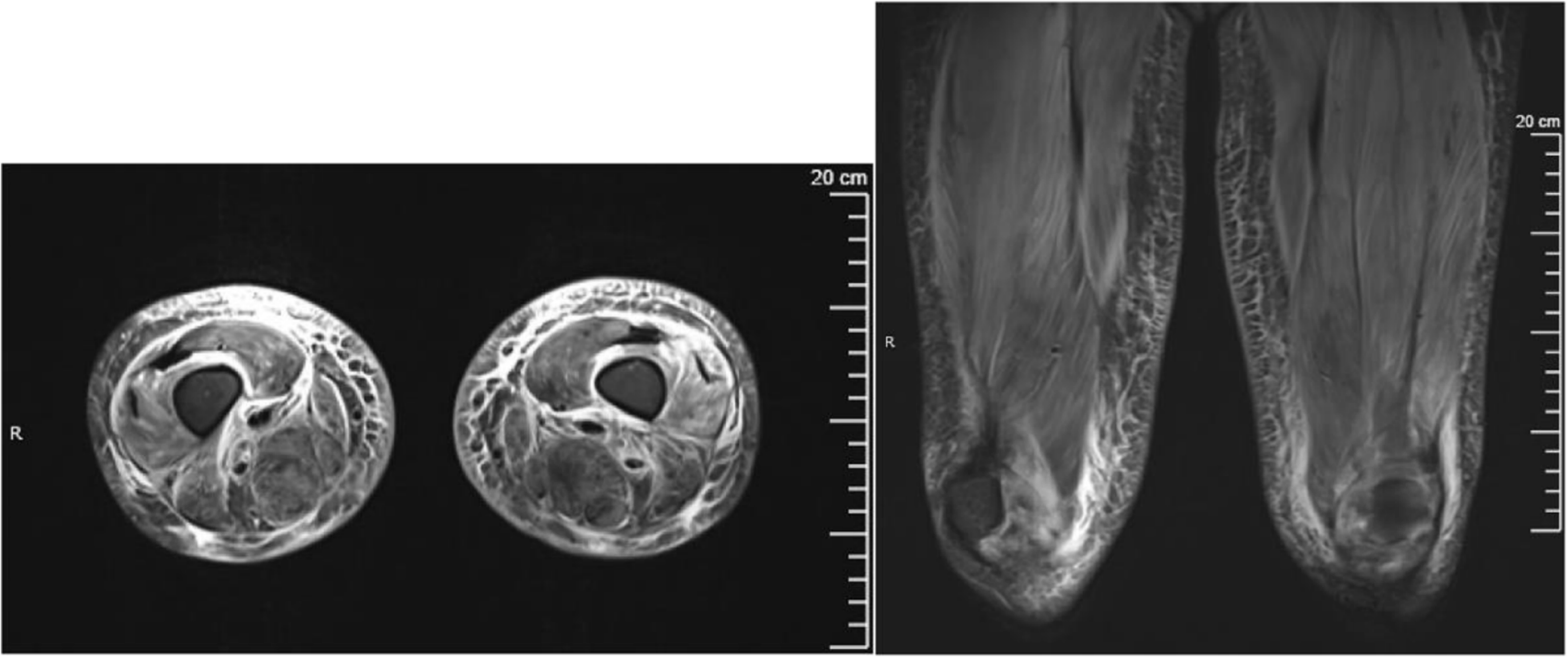
MRI Case 1: Axial and coronal STIR sequences demonstrating diffuse bilateral muscle edema, fasciitis, and subcutaneous edema

Laboratory workup is summarized in Table 1. Results were significant for the following: CK 2952 IU/L, AST 394 IU/L, ALT 101 IU/L, LDH 865 IU/L and aldolase 35 U/L. Her inflammatory markers were elevated, and she had hypoalbuminemia without significant proteinuria. Her myositis-specific antibodies and myositis-associated antibodies were negative (Oklahoma Medical Research Foundation). Imaging results are summarized in Table 2. MRI of the proximal musculature was notable for diffuse myositis, fasciitis, and subcutaneous edema consistent with inflammatory myositis (Fig. 1). Swallow study was concerning for severe pharyngeal weakness. A muscle biopsy was performed and showed perifascicular pattern of muscle atrophy, basophilic degeneration, MHC class I expression, and complement deposition, consistent with JDM. This diagnosis was further supported by her clinical findings, laboratory workup and imaging results.

**Table 1:** Summary of select results from laboratory studies at time of presentation. Bold indicates abnormal values

	*Ref ranges*	Case 1	Case 2
CK (IU/L)	*81–279*	**2952**	20
Aldolase (U/L)	*3.4–8.6*	**35**	4.7
AST (IU/L)	*18–57*	**394**	45
ALT (IU/L)	*2–30*	**101**	25
LDH (IU/L)	*188–403*	**865**	281
Albumin (g/dL)	*3.6–4.7*	**2.6**	**2.4**
Creatinine (mg/dL)	*0.25–0.94*	0.29	0.23
BUN (mg/dL)	*7–18*	15	11
Fecal calprotectin (mcg/g)	*< 120*	86.5	N/A
ESR (mm/hr)	*0–20*	15	10
CRP (mg/dL)	*0–0.8*	0.3	< 0.3
ANA	*< 1:40*	1:40	**1:80**
C3 (mg/dL)	*89–173*	**72.2**	**61**
C4 (mg/dL)	*15–45*	11.3	19.7
UA	*N/A*	Normal	Normal
Urine protein/ creatinine	*< 0.2*	**0.6**	0.1
TSH (uIU/dL)	*0.70–5.97*	4.51	1.11
fT4 (ng/dL)	*0.96–1.77*	N/A	1.1
vWF Ag (%)	*Dependent on blood type*	115	**> 580**
Serum Neopterin (nmol/L)	*< 10*	**22.8**	9.2
Ferritin (ng/mL)	*11–320*	**747**	27

**Table 2:** Summary of imaging results at time of presentation

	Case 1	Case 2
MRI	Diffuse myositis, fasciitis, subcutaneous edema, muscle atrophy, fatty infiltration	Diffuse edema involving subcutaneous tissues and musculature
CXR	No pulmonary edema	Bilateral pleural effusions and pericardial effusion
Echo	Normal	Pericardial effusion, mild RA diastolic collapse
Abd US	Largely normal (mildly echogenic liver but normal Doppler)	Normal organs, mild ascites and perihepatic and perisplenic free fluid
Other	Swallow study with severe pharyngeal dysphagia	None

Treatment was initiated with with 30 mg/kg/day (pulse-dose) of IV methylprednisolone (IVMP) × 3 days followed by 30 mg (0.5 mg/kg) oral prednisone, 2 g/kg intravenous immunoglobulin (IVIG), and 20 mg subcutaneous methotrexate (15 mg/m^2^) and 1 g of rituximab (750 mg/m2 up to 1 g). Her edema was managed with albumin infusions followed by furosemide. Due to pharyngeal weakness, a nasogastric tube was placed for feeding. Her hospital course was complicated by acute hypotension with widened blood pressures during IVMP infusion. This was thought to be secondary to a medication reaction versus intravascular depletion, and it resolved shortly after receiving a 5% albumin infusion. She required no vasoactive support. She was also briefly transferred to the PICU due to concern for impending respiratory failure given bulbar weakness as evidenced by dysphonia, dysphagia, and poor head control, but she ultimately did not require respiratory support.

Her weakness, edema and rash improved during her hospitalization. She was discharged home two weeks later on 30 mg prednisone daily, 20 mg subcutaneous methotrexate weekly, 1 g IVMP twice weekly, 2 g/kg IVIG monthly, and a second dose of rituximab (1 g IV) which was given 18 days after the first dose. She has shown slow clinical improvement over the past two years, however, her treatment has been complicated by non-adherence to medications. She is currently receiving 1 g of IVMP every month, 1 g/kg of IVIG every 2 months, 10 mg (0.14 mg/kg) of prednisone daily and 20 mg of subcutaneous methotrexate weekly. At her most recent physical therapy assessment and office visit, 2 months prior to the time of writing, her MMT8 score was 80/80 and her CMAS score was 50/52. Her muscle enzymes have fluctuated with an overall downtrend, and they have been normal for the past 3 months. Her rash has completely resolved. Although her course was severe, she has no cutaneous stigmata of refractory disease, such as calcinosis or lipodystrophy.

### Case 2

A 4 year-old previously healthy girl was transferred to our PICU from an outside medical center ED for workup and management of generalized anasarca, manifesting as diffuse edema, pleural and pericardial effusions, and abdominal ascites.

In the four months prior to admission, her mother noted that she had increasing abdominal distension and facial edema, as well as cough, shortness of breath, and orthopnea. Other symptoms included progressive weakness, fatigue, abdominal pain, and a 5–10 lb. weight gain. She had been evaluated by multiple providers in the ED as well as her PCP due to these symptoms; however, no etiology was identified, and she was ultimately diagnosed with a viral syndrome. Due to worsening abdominal pain and increasing work of breathing, her mother brought her back to the outside hospital ED.

In the outside hospital ED, she had a Chest X-ray which showed bilateral pleural effusions and a pericardial effusion. Echocardiography and Neck/Chest/Abdomen CT confirmed the presence of large pericardial and pleural effusions and abdominal ascites (Table 2). She required transfer to the outside hospital PICU shortly after admission, where she was intubated secondary to increasing shortness of breath. Pericardiocentesis was performed, and 150 mL of fluid was removed. A pericardial drain was placed as well as bilateral chest tubes. Extensive workup at this time was largely unrevealing from an infectious disease, rheumatologic, and oncologic perspective (including lymph node biopsy and evaluation of the pericardial and pleural fluid). MRI, however, showed diffuse edema of the musculature.

Her respiratory status improved, so she was extubated and transferred to our institution for ongoing evaluation. On examination, she had diffuse non-pitting edema of all four extremities, facial edema and significant abdominal distension as well as a small aphthous ulcer on her tongue and lymphadenopathy in her cervical chain and bilateral axillae. She was noted to have a wide based gait, some difficulty rolling over, and inability to stand without using her hands to help pull herself up. Her significantly increased abdominal girth likely contributed to her inability to perform activities that required core strength. Full muscle testing was unable to be obtained due to cooperation. No muscle or bony tenderness, joint pain or swelling, or rash were appreciated. Vital signs, including heart rate, blood pressure, temperature, and SpO2 were in normal range for age. Laboratory workup is summarized in Table 1 and is notable for normal muscle enzymes. Repeat MRI revealed diffuse edema of subcutaneous tissues and musculature. Muscle biopsy showed perimysial and perivascular lymphocytic inflammation, consistent with inflammatory myositis, and HLA staining showed significant HLA Class I upregulation consistent with JDM. A diagnosis of juvenile dermatomyositis was made based on her imaging and pathology findings. Myositis specific antibody panel was sent, which was notable only for weakly positive Ro60. Notably, at this time, she did not have the classic clinical manifestations of rash nor the typical laboratory values consistent with myositis, such as elevated CK, ALT, LDH, or aldolase. Furthermore, it was difficult at the time of presentation to differentiate weakness from deconditioning and limitation secondary to enlarged body habitus secondary to edema.

During her hospital course, she experienced reaccumulation of her pleural and pericardial effusions after her drains were removed, requiring repeat drain placement. She was initially treated with furosemide, indomethacin, and colchicine for her refractory edema. Once the pathology results returned consistent with JDM, treatment was initiated with pulse dose (30 mg/kg/day) of IVMP. She had no significant improvement after 5 days of pulse dose IVMP, so 2 g/kg IVIG was administered over 2 days (1 g/kg/day × 2) Two days later, she was noted to have marked improvement clinically in terms of improvement in her edema as well as a decrease in her inflammatory markers. She was discharged shortly thereafter with a plan for 30 mg/kg IVMP every week, 2 g/kg IVIG every 3 weeks, 30 mg (1 m/kg) prednisone daily, and 15 mg methotrexate weekly (15 mg/m^2^).

Approximately 2 weeks after discharge, this patient was readmitted to our hospital with significant weakness and re-accumulation of pleural and pericardial effusions and abdominal ascites. At this point, she did display the classic Gottron’s papules consistent with JDM. She was treated with 2 g/kg IVIG and 600 mg of rituximab (750 mg/m2), with improvement in her symptoms. She received another dose of rituximab 3 weeks later as an outpatient. It was delayed by one week due to the initial denial from the insurance company, which was approved on appeal. Over the next several months, she continued to have frequent readmissions for weakness, muscle pain, and recurrent effusions, requiring an increase in frequency of IVIG to 2 g/kg every 2 weeks, and escalating steroids to 30 mg (1 mg/kg) prednisone daily and 30 mg/kg IVMP weekly. Her weekly IVMP was continued for four months after discharge. Oral prednisone was tapered over 7 months after discharge. She continued biweekly IVIG for three more months until the fall of 2020 (Mom was anxious to bring the patient to an infusion center due to a local rise in COVID cases, so treatment was interrupted). IVIG was re-initiated in the spring of 2021 secondary to disease flare as evidenced by weakness and elevated inflammatory markers. Steroids were not restarted. Her current medication regimen includes 2 g/kg IVIG every 3 weeks and 15 mg subcutaneous methotrexate weekly. She has received no additional doses rituximab.

This patient continues to exhibit core and upper extremity weakness, but she no longer has edema or reaccumulating effusions. Family is non-compliant with physical therapy, which likely contributes to her continued weakness and deconditioning. She has no evidence of calcinosis or lipodystrophy.

## Discussion

Juvenile Dermatomyositis is a chronic multisystem inflammatory disease characterized primarily by rash and myositis; however, other systems are often affected, including the heart, lungs, and GI tract. Generalized edema (i.e. anasarca) is a very rare manifestation of JDM, and is likely attributable to dysfunction of the vasculature [14]. Periorbital edema and limb edema are well-known associations with JDM, but generalized anasarca has been rarely reported, even in the adult literature on Dermatomyositis (DM) [15–20]. A summary of the pediatric patients in the English literature over the past 20 years with juvenile dermatomyositis can be found in Table 3. Without proper identification and treatment, anasarca can be life-threatening, as seen in our two patients, both of whom required ICU-level management.

**Table 3:** Review of reported cases of juvenile dermatomyositis patients presenting with anasarca from 2001 to 2021

Authors	Age	Sex	Pertinent medical history	Muscle weakness	Rash	Studies that supported diagnosis	Major complications	Treatment	Outcome
Mehndiratta et al	8 yo	F	Progressive deformity of elbows and knees	+	–	Elevated muscle enzymes, muscle biopsy, EMG	Mucosal ulcerations, dysphagia, GI bleed	Prednisolone	Death due to septicemia and DIC
Saygi et al	4 yo	M	Similar episode 6 months prior, resolved spontaneously; recent URI	+	+	Elevated muscle enzymes, EMG, MRI	–	Prednisolone, methotrexate	Improvement in symptoms
Jimenez et al	13 yo	F	Previously healthy	+	–	Elevated muscle enzymes, muscle biopsy, EMG	–	IVMP, prednisolone, IVIG, cyclophosphamide, chloroquine, methotrexate	Improvement in symptoms
Wakhlu et al	3.5 yo	M	Previously healthy	+	–	Elevated muscle enzymes, muscle biopsy	–	Prednisolone, methotrexate	Improvement in symptoms
Sharma et al	8 yo	M	Previously healthy	+	–	Elevated muscle enzymes, MRI	–	IVMP, prednisolone, methotrexate	Improvement in symptoms
Shelley et al	8 yo	M	Previously healthy	+	+	Elevated muscle enzymes, EMG, muscle biopsy, myositis antibodies (Jo-1 IgG, anti-Mi2)	Hypotension requiring ICU level care and catecholamine support (dopamine, norepinephrine)	IVMP, prednisolone, IVIG, azathioprine	Improvement in symptoms
Zedan et al	3.5 yo	M	Recent URI	+	+	Elevated muscle enzymes, EMG, MRI	–	IVMP, prednisolone, IVIG, azathioprine	Improvement in symptoms
Mitchell et al	7 yo	F	Previously healthy	+	+	Elevated muscle enzymes, elevated factor VIII related antigen, EMGs, MRI	–	IVMP, prednisolone, methotrexate, IVIG, hydroxychloroquine	Improvement in symptoms
Karabiber et al	14 yo	M	Previously healthy	+	+	Elevated muscle enzymes, EMG, MRI	–	IVMP, prednisolone	Improvement in symptoms
Chandrakasan et al	4 yo	M	Recent URI (parvovirus PCR positive)	+	+	Elevated muscle enzymes, EMG, MRI	–	IVMP, prednisolone, methotrexate	Improvement in symptoms
Nickavar et al	7 yo	M	Initially treated for nephrotic syndrome	+	+	Elevated muscle enzymes, EMG	Seizures, renal failure (creatinine 8, renal biopsy with FSGS), pulmonary edema requiring hemodialysis	IVMP, IVIG, plasmapheresis	Unknown

The Peter and Bohan criteria that were established in 1979 are still widely used by pediatric rheumatologists to establish the diagnosis of JDM [21]. These criteria include proximal muscle weakness, elevated muscle enzymes, characteristic rashes, and a muscle biopsy and/or EMG consistent with JDM. In classic presentations with the aforementioned three characteristics, it has become acceptable to replace the last two more invasive criteria with MRI findings of myositis in the proximal musculature. Newer classification criteria reflect this change in practice [22–24]. Although myositis specific antibodies can also be helpful in making the diagnosis of JDM, only one case in the literature of a JDM patient presenting with anasarca was reported to have a positive myositis antibody [6].

The second case presentation had neither the classic symptoms nor the typical laboratory findings associated with JDM at the time of diagnosis. Her presentation of florid anasarca, with pleural and pericardial effusions and abdominal ascites, preceded onset of weakness and rash by several weeks. This illustrates the importance of recognizing that anasarca can be either a presenting feature or later manifestation of JDM, even in the absence of other typical signs and symptoms.

The differential diagnosis for anasarca is broad and includes oncologic disease, hematologic abnormalities such as DVT, infectious diseases, hypothyroidism, cardiac failure, liver cirrhosis, nephrotic syndrome, malabsorption, and many others, including idiopathic capillary leak syndrome [25, 26] (Table 4). In JDM, increased capillary permeability is proposed to be secondary to complement activation and immune-complex deposition, leading to microischemia and subsequent disruption of the vascular endothelium. Loss of protein into the extravascular space leads to decreased oncotic pressure and promotes movement of intravascular fluid into the extravascular space, resulting in edema, which can be quite extensive [8, 17]. Interestingly, both of our patients initially presented with low C3 levels. Complement activation is thought to be a major component of JDM, and complement deposition has been found in the vessels of skeletal muscle in children with this disease [27, 28]. We may posit that complement activation and deposition in active disease may account for the low C3 that was seen in our patients. Indeed hypocomplementemia in JDM has been described in the literature [29]. However, hypocomplementemia was not a defining feature of the other cases of JDM-associated anasarca that were reviewed, although it may not have been checked in these cases. Increased serum concentration of Factor VIII (von Willebrand Factor Ag) has been noted in the serum of some patients with JDM who develop anasarca compared to patients with JDM who do not develop this complication [8, 30, 31]. FVIII is released by endothelial cells and is a marker of vascular damage, and is often used to trend disease activity in JDM [31]. Our second patient had extremely high levels of FVIII during her initial hospitalization, although our first patient’s levels remained normal.

**Table 4:** Overview of different causes of generalized edema, separated based on pathophysiology, including examples of each process

Increased hydrostatic pressure	Decreased intravascular oncotic pressure	Increased vascular permeability	Increased extravascular oncotic pressure
Heart failure	Nephrotic syndrome	Toxic shock syndrome	Lymphatic obstruction
Liver disease	Malnutrition (e.g. kwashiorkor)	Idiopathic capillary leak syndrome	
Venous obstruction (e.g. thrombus)	Protein-losing enteropathy		

The presence of anasarca in JDM may be a predictor of more severe disease activity and poorer outcomes [1, 8, 19]. Our patients had highly active disease and required more aggressive and prolonged immunosuppression than typical patients with JDM. IVIG has been shown to be the most effective treatment in patients with severe or refractory JDM, with increased doses of up 2 g/kg every 2 weeks being required to treat the disease [32]. This was indeed the case with both of our patients, particularly the second patient, who did not start to improve until after the initiation of IVIG.

## Conclusion

Anasarca is an unusual presenting feature of JDM; however, it should be considered in patients with edema of unknown etiology. Muscle enzymes are usually elevated in JDM, but normal enzymes and absent skin findings does not exclude JDM as a possible etiology for edema. MRI should be obtained to evaluate for myositis, and muscle biopsy should be pursued to confirm the diagnosis if there is a high level of clinical concern. In the literature, patients with JDM who either present with anasarca or develop anasarca during the course of their disease have a very refractory course, requiring aggressive immunosuppression. Doses of IVIG up to 2 g/kg every 2 weeks have been necessary, as well as adjunctive treatment with biologic or cytotoxic agents, such as rituximab and cyclophosphamide, respectively. Early identification and treatment of these patients is important and may lead to significantly improved outcomes.

## Data Availability

The datasets generated and/or analysed during the current study are not publicly available due to HIPAA compliance but are available from the corresponding author on reasonable request.

## Abbreviations

JDM: Juvenile Dermatomyositis
DM: dermatomyositis
IVIG: intravenous immune globulin
IVMP: intravenous methylprednisolone
CT: computed tomography
ICU: intensive care unit
CK: creatine kinase
AST: aspartate aminotranferase
ALT: alanine transaminase
LDH: lactate dehydrogenase
RF: Rheumatoid Factor
DVT: deep venous thrombosis
HLA: human leukocyte antigen
MRI: magnetic resonance imaging
PICU: pediatric intensive care unit
ED: emergency department
PCP: primary care physician
MHC: major histocompatibility complex
MMT8: Manual Muscle Test 8
CMAS: Childhood Myositis Assessment Scale
